# Visualisation of PCNA Monoubiquitination *In Vivo* by Single Pass Spectral Imaging FRET Microscopy

**DOI:** 10.1371/journal.pone.0009008

**Published:** 2010-02-02

**Authors:** Christopher Batters, Hannah Zhu, Julian E. Sale

**Affiliations:** 1 Medical Research Council Laboratory of Molecular Biology, Cambridge, United Kingdom; 2 Medical Research Council Laboratory of Molecular Biology, Division of Protein & Nucleic Acid Chemistry, Cambridge, United Kingdom; University of Minnesota, United States of America

## Abstract

Monoubiquitination of the DNA sliding clamp, PCNA, plays a central role in the control of damage bypass during replication. By combining a widely-spaced FRET donor/acceptor pair (CFP and mRFP) with spectral imaging, we have developed a simple method for the visualisation of PCNA monoubiquitination in both fixed and live cells with a single imaging pass. We validate the method with genetic controls in the avian cell line DT40 and use it to examine the intracellular dynamics of PCNA ubiquitination following subnuclear UV irradiation. This general approach is likely to be of utility for live imaging of post-translational modifications of a wide range of substrates *in vivo*.

## Introduction

The monoubiquitination of the DNA sliding clamp, PCNA, plays a central role in the control of translesion synthesis in eukaryotes [1], [2], [3], [4]. Conjugation of ubiquitin to lysine 164 of PCNA is induced by the arrest of replication by DNA damage. It is mediated by the E3 ubiquitin ligase RAD18 in conjunction with the E2 ubiquitin conjugating enzyme RAD6, although in higher eukaryotes there is evidence that other enzymes can be used as well [5], [6]. The presence of ubiquitin on PCNA increases the affinity of the clamp for members of the Y-family of specialised DNA polymerases, which contain ubiquitin binding motifs, that are able to directly bypass DNA damage in a process known as translesion synthesis [7]. A single ubiquitin on PCNA can also act as a ‘seed’ for the formation of a non-canonical K63-linked polyubiquitin chain mediated by the E3 ligase RAD5 acting in concert with UBC13/MMS2 in yeast [2], or by the RAD5 homologues SHPRH/HLTF in vertebrates [8], [9], [10], [11]. Formation of these chains is linked to a recombinational mode of bypass, sometimes termed template switching, that may also employ the helicase activity of RAD5 [12].

Ubiquitin can be removed from PCNA by the ubiquitin-specific protease USP1 [13], although blockade of this step does not appear to have a significant impact on the DNA damage response [14]. Further, it appears from Western blotting studies in human cells that ubiquitin-PCNA conjugates persist on chromatin for many hours after the removal of DNA damage, suggesting a model in which PCNA ubiquitination acts to promote bypass behind the replication fork [15]. In agreement with this, our own studies in the chicken cell line DT40 have shown that PCNA ubiquitination is required for post-replicative gap filling, but not for maintenance of fork progression on a damaged DNA template [16]. However, PCNA ubiquitination in DT40 appears to be rather more transient than in the human cell lines studied. DNA damage results in only a modest accumulation of conjugates as monitored by Western blotting unless USP1 is disrupted, in which case much higher steady state levels are observed [5], [14].

Western blotting is currently the only method available for monitoring PCNA ubiquitination and is, by its very nature, rather crude as it only provides an indication of the average level of the modification in a large number of cells. Much has been learned about the subcellular dynamics of DNA repair proteins by studying their localisation within cells using fluorescence microscopy. However, such studies of PCNA ubiquitination have been hampered by the lack of an antibody specific for the ubiquitinated species of PCNA. A potential solution to this problem exists in the form of Förster Resonance Energy Transfer (FRET) [17]. FRET occurs when two fluorophores are brought into very close proximity (less than 10 nm). Under these conditions, emission energy from the donor can be directly, and non-radiatively, transferred to the acceptor fluorophore resulting in emission of photons by the acceptor. FRET is a widely used technique in biology that allows the determination of interactions between two proteins of interest within the range of 3–10 nm, thus allowing discrimination between simple co-localisation within the limits of the optical resolution of light microscopes and genuine interaction.

Three basic classes of method exist for detecting FRET [17]. 1. Fluorescence lifetime imaging monitors the reduction in the fluorescent lifetime of the donor fluorophore that occurs upon energy transfer. This technique requires specialist equipment, including expensive femtosecond lasers, and can be limited in the complex *in vivo* environment by uncertainty over the identity of the molecules to which energy is transferred. 2. Acceptor photobleaching monitors the emission intensity of the donor molecule before and after destruction of the acceptor fluorophore. This technique is quantitative and specific but necessarily destructive and is not well suited to long-term live cell imaging. 3. Sensitised emission monitors the indirect excitation of a donor fluorophore by energy transfer. Although conceptually straightforward and attractive in principle, the implementation of this technique for live cell imaging is complicated by the requirement for the acquisition and registration of multiple control images from different specimens.

Spectral imaging refers to the ability to acquire full spectral detail from an image rather than inferring colour from the more traditional use of filters to separate blocks of spectral information onto single detectors. Use of spectral imaging for monitoring FRET has a number of advantages as the intensities of the donor and acceptor can be simultaneously measured allowing ratiometric detection of FRET [18], [19], [20], [21].

Here we report the detection of PCNA ubiquitination in both fixed and live cells through the use of spectral imaging to directly monitor FRET between fluorescently tagged ubiquitin and PCNA. We employ the rather under appreciated FRET pair cyan fluorescent protein (CFP) and monomeric red fluorescent protein (mRFP) [22], and confirm that energy transfer between them is comparable to the more commonly used pair, CFP and YFP. We demonstrate FRET between CFP-ubiquitin and mRFP-PCNA using genetic controls in the avian cell line DT40. By irradiating cells with UV light through a 3 µm microporous membrane to induce localised damage, we demonstrate a marked difference in the kinetics and extent of PCNA modification between cells irradiated in S phase and in G1 phase of the cell cycle.

## Results

### CFP and mRFP Are a Widely Spaced, Yet Efficient FRET Pair

**Figure 1:**
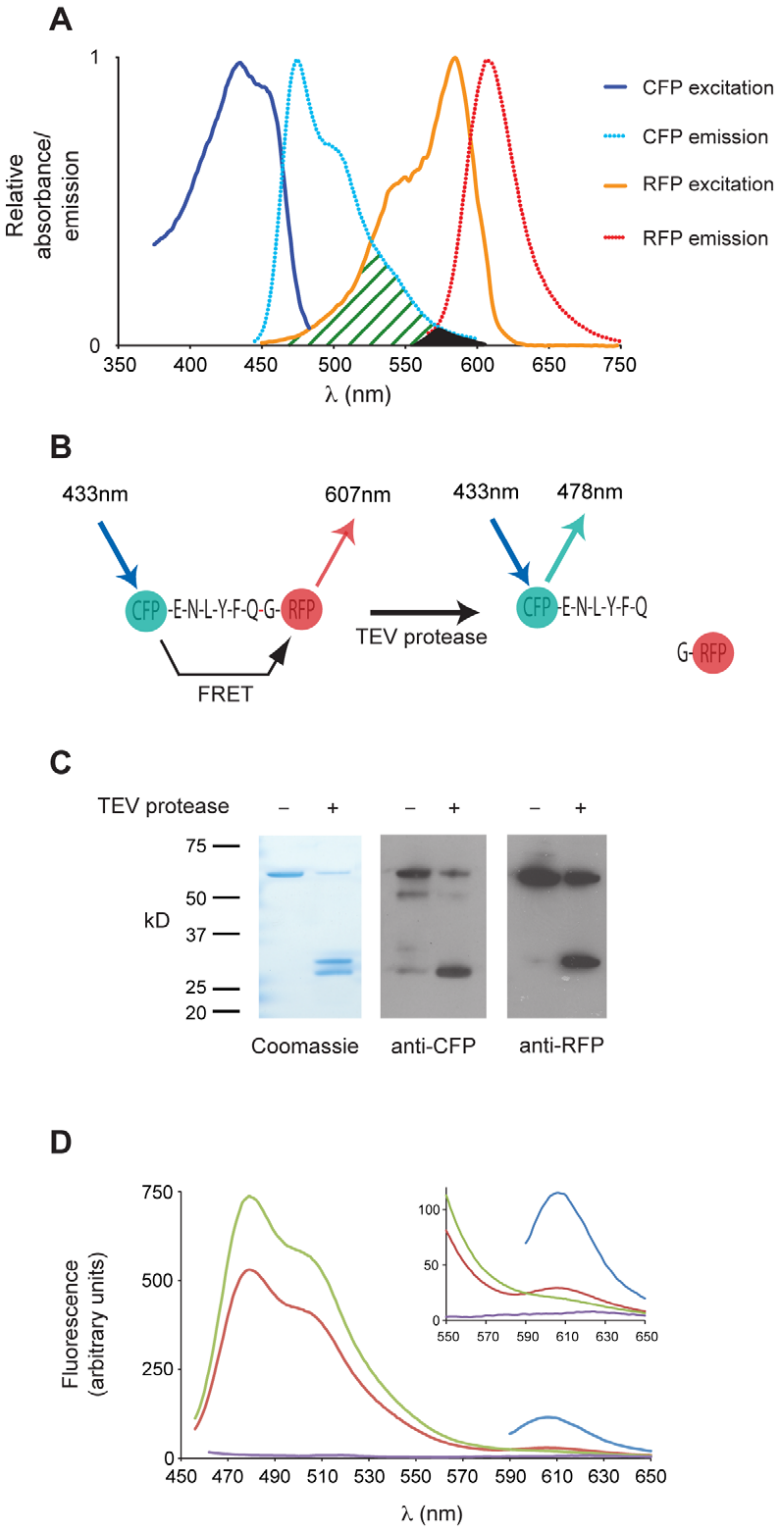
FRET between CFP and mRFP in solution. A. Absorbance and emission spectra of CFP and mRFP. The spectra are normalised to the excitation or emission maximum. The overlap (Jλ) between CFP emission and mRFP excitation is shown in green. The overlap in the emission spectra is shown in black. B. Schematic of the CFP-mRFP fusion protein. Before cleavage with TEV protease FRET can take place between CFP and mRFP resulting in energy from the excited CFP being transferred to the mRFP, diminishing the direct emission by CFP at 478 nm and stimulating emission of RFP at 607 nm. Following cleavage, energy transfer is lost resulting in an increase in 478 nm emission by CFP. C. Cleavage of the mRFP-TEV-CFP fusion protein by TEV protease. An aliquot of the reaction was taken from the spectrophotometer at the point at which the CFP emission peak had plateaued following addition of TEV protease. Aliquots were run on SDS-PAGE, stained with Coomassie Blue and transferred for Western blotting with anti-CFP and anti-RFP. D. FRET between CFP and mRFP. Spectra of the CFP-TEV-mRFP fusion protein before (red line) and after (green line) cleavage with TEV protease. The purple line shows the blank control and blue line the emission of the mRFP component of the fusion protein when directly excited at 584 nm.

The most commonly employed fluorescent proteins in FRET studies are the spectrally shifted variants of green fluorescent protein, CFP (cyan) and YFP (yellow) [23]. However, use of the CFP/YFP pair requires extensive controls to correct for the spectral overlap of the CFP and YFP emission spectra. In general, more widely spaced fluorophores will not undergo efficient FRET since the efficiency of FRET is dependent on the overlap between the emission spectrum of the donor and acceptor spectrum of the acceptor (Jλ) [24]. A further consideration is that many red fluorescent proteins e.g. DsRed exhibit strong tetramerisation, which limits their utility as protein tags. Monomeric RFP is a derivative of DsRed that exists as a monomer and exhibits very rapid maturation (<1 hour) [22]. The excitation spectrum of monomeric RFP (mRFP) has a significant shoulder at shorter wavelengths, which overlaps well with the emission spectrum of CFP (Figure 1A, shaded green area). Equally, the overlap of the emission spectra of CFP and mRFP is minimal (Figure 1A, black region). To measure the FRET efficiency between CFP and mRFP we constructed a fusion protein in which CFP was linked to mRFP via a short sequence containing a cleavage site for the Tobacco Etch Virus (TEV) protease (Figure 1B). The emission spectrum of a solution of the CFP-mRFP fusion protein excited at 433 nm was determined before, and 2 hours after, the addition of TEV protease. The fusion protein was cleaved efficiently to yield CFP and mRFP (Figure 1C). Cleavage resulted in an increase in fluorescence at the CFP peak of 38.6% with a corresponding diminution in fluorescence at 607 nm of 32%. This corresponds to a FRET efficiency of 0.28. This agrees closely with another recent determination of the FRET efficiency between CFP and mRFP of 0.25 [25]. Thus, CFP and mRFP are an efficient FRET pair that offer a number of potential advantages in their spectral properties compared with CFP and YFP.

### Detection of FRET between CFP-Ubiquitin and mRFP-PCNA in Fixed Cells by Acceptor Photobleaching

**Figure 2:**
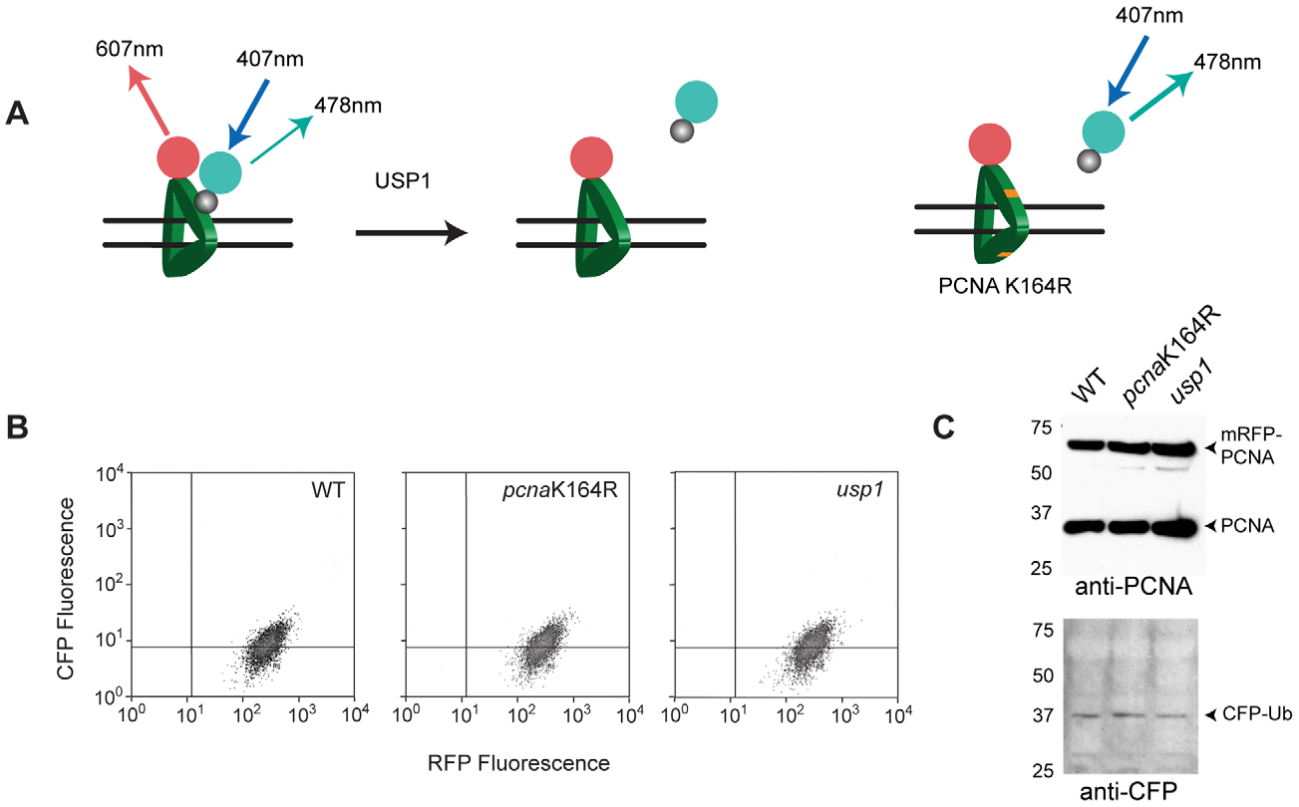
Expression of fluorescently tagged PCNA and ubiquitin in DT40 cells. A. The genetics of PCNA monoubiquitination. PCNA is monoubiquitinated at lysine 164 by RAD6/RAD18. The monoubiquitin can be cleaved from PCNA by USP1. Thus, when ubiquitin is conjugated to PCNA, FRET may take place between the CFP attached to ubiquitin and mRFP attached to PCNA. No FRET should be seen in a *pcna*K164R cell line and the signal should be exaggerated in a *usp1* line. B & C Expression of CFP-ubiquitin and mRFP-PCNA in DT40. B. FACS plots. C. Western blots.

In order to study PCNA ubiquitination by FRET we expressed ubiquitin tagged at its N-terminus with CFP and PCNA tagged at its N-terminus with mRFP in chicken DT40 cells. Previous work has demonstrated that both ubiquitin and PCNA tagged with fluorescent proteins are able to participate in normal cellular functions [26], [27]. The genetically tractable DT40 cell line [28] provides a robust genetic platform to test the detection of FRET resulting from PCNA ubiquitination as mutants are available both in which PCNA cannot be ubiquitinated (*pcna*K164R) [5], [29] and in which the hydrolysis of ubiquitin from PCNA is inhibited (*usp1*) [5], [14] (Figure 2A). For each of these mutants and for wild type cells we established clones expressing CFP-ubiquitin, mRFP-PCNA or both. In the case of the *pcna*K164R mutant we expressed mRFP-*pcna*K164R. Clones were matched for expression of each component using selection by cell sorting and this was confirmed by cytometry and by Western blotting (Figure 2B & C). Relatively low levels of stable expression were selected to avoid a concentration-dependent FRET signal.

**Figure 3:**
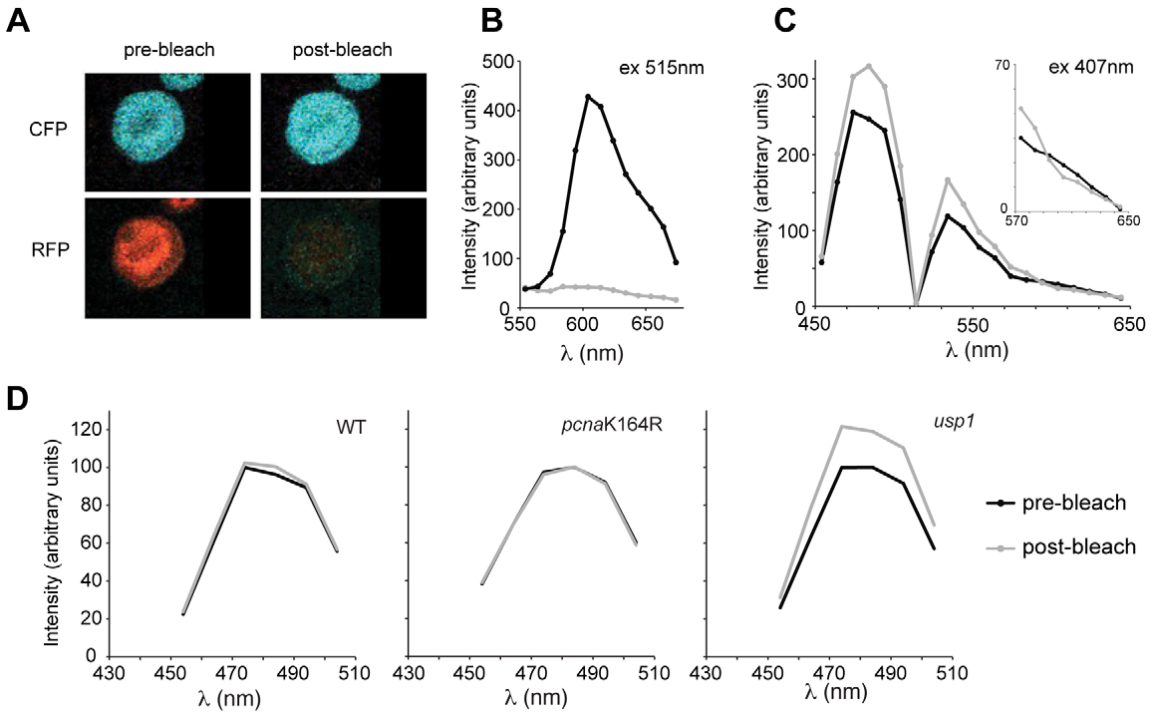
Detection of PCNA ubiquitination by acceptor photobleaching FRET. A. A *usp1* DT40 cell before and after bleaching of the mRFP acceptor. B. Whole cell spectrum, of the cell shown in A, with excitation at 515 nm (Ex515/ΣλmRFP) before (black line) and after (grey line) photobleaching. C. Whole cell spectrum with excitation at 407 nm (Ex407/ΣλCFP) before and after photobleaching. Inset panel shows a zoomed in region covering the wavelengths around the mRFP emission maximum at 607 nm. D. Ensemble averages from 30 cells of the indicated genotype before and after photobleaching of mRFP. Intensity was normalised to pre-photobleach maximum.

To obtain initial evidence of FRET between CFP-Ub and mRFP-PCNA *in vivo* we employed acceptor photobleaching to monitor the increase in the emission of the CFP donor fluorophore following destruction of the acceptor mRFP by exposure to high intensity excitation. This effect is clearly seen in the *usp1* cell shown in Figures 3A. Following bleaching of acceptor fluorescence the intensity of the CFP signal increases (Figure 3A–C). To compare the level of Ub-PCNA FRET in wild type, *usp1* and *pcna*K164R cells, we monitored CFP intensity following mRFP photobleaching in >30 individual cells for each line (Figure 3D). A small increase in CFP signal was seen in wild type cells following photobleaching, while no change was seen in the *pcna*K164R mutant. A more substantial increase in CFP signal was seen in *usp1* cells. This is consistent with previous Western blot data in DT40, which reveals a low steady state of ^Ub^PCNA in undamaged DT40 cells [5].

### Direct Detection of PCNA-Ubiquitination by Spectral Imaging of Sensitised Emission FRET

Although acceptor photobleaching is a robust method for determining FRET, it is destructive and only suitable for imaging fixed cells and, in this respect, detection of FRET by sensitised emission is preferable. By allowing simultaneous microscopy and spectroscopy, spectral imaging can facilitate ratiometric FRET detection [19] and is now widely available on a number of microscopy platforms.

**Figure 4:**
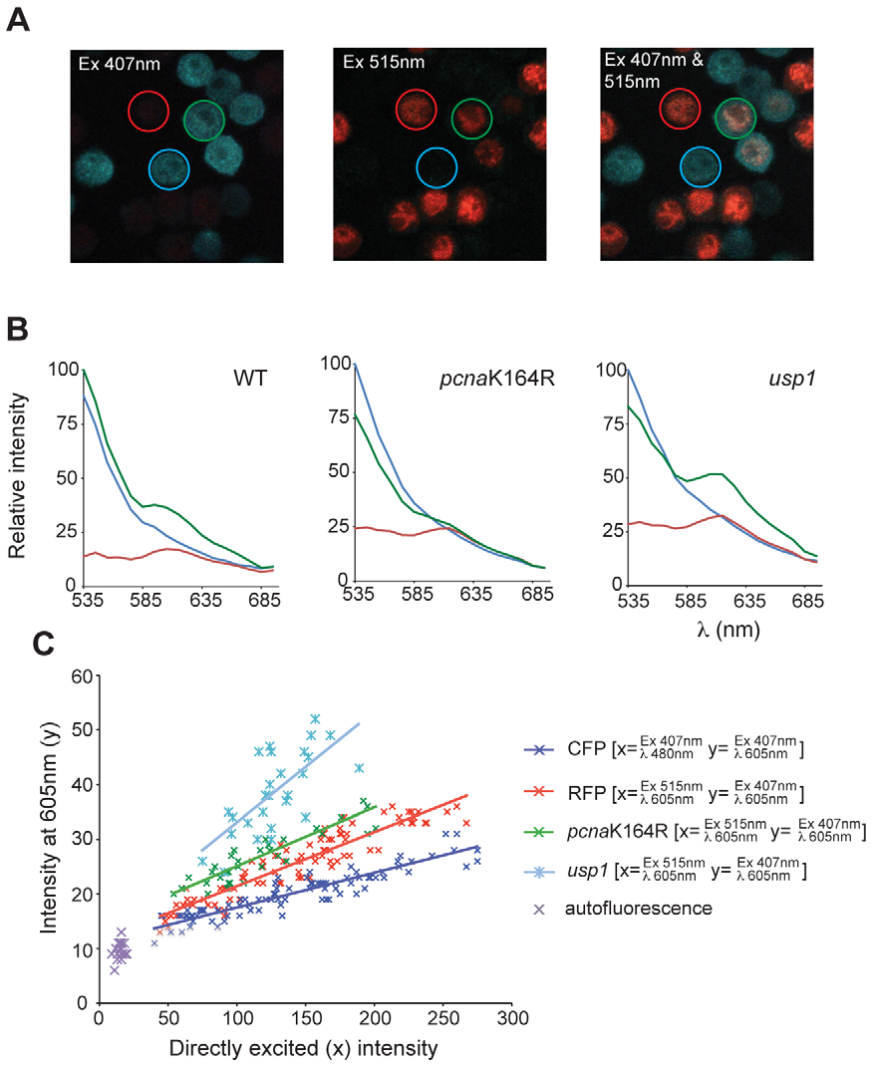
Direct visualisation of PCNA ubiquitination by spectral imaging of fixed DT40 cells. A. Example images of cell mixtures with regions of interest, red circle  =  cell expressing mRFP-PCNA only, blue circle  =  CFP-ubiquitin only and green circle  =  both. B. Ensemble average spectra from cells expressing mRFP-PCNA and/or CFP-ubiquitin. Key as above. The emission maximum of mRFP is 607 nm. C. Relationship of directly excited intensity of a fluorophore to its potential, indirect, contribution to the FRET signal (see text).

For each DT40 line (WT, *pcna*K164R and *usp1*), clones expressing CFP-Ub and/or mRFP-PCNA (or mRFP-*pcna*K164R as appropriate) were mixed on a slide, fixed and visualised with excitation at 407 nm or 515 nm over a range of wavelengths from 535 nm to 685 nm. Example images are shown in Figure 4A with regions of interest indicating a cell expressing CFP-Ub only (blue), mRFP-PCNA only (red) and both (green). Spectral data was collected from >50 whole cells of each expression pattern for each genotype, averaged and normalised to the CFP maximum. A FRET signal is expected at 607 nm, the emission maximum of mRFP [22]. However, this raw FRET signal [RawFRET^λ607^] will also contain spectral bleedthrough from CFP [CFP^Ex407/λ607^] and from mRFP directly excited by the 407 nm blue diode laser [mRFP^Ex407/λ607^]. Thus, the actual FRET signal at pixel (x, y) [FRET^λ607^_(x,y)_] is given by:
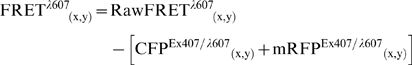



The CFP^Ex407/λ607^ and mRFP^Ex407/λ607^ components are clearly visible in the cells expressing only CFP-Ub or mRFP-PCNA (Figure 4B, blue and red lines, respectively). In wild type cells an additional signal is present when both CFP-Ub and mRFP-PCNA are expressed (Figure 4B, green line). This signal is lost in the *pcna*K164R mutant and exaggerated in the *usp1* cells, supporting it being RawFRET^λ607^. By examining cells expressing a range of levels of CFP-Ub and mRFP-PCNA, the contributions of [CFP^Ex407/λ607^] and [mRFP^Ex407/λ607^] to the raw FRET signal can be shown to be a linear function of the intensity of each fluorophore excited directly over the range of fluorophore concentrations present (Figure 4C, blue and red points respectively. The FRET signal in *usp1* cells is clearly distinguishable (Figure 4C, light blue line) when compared with *pcna*K164R cells (Figure 4C, green line). Together, these data demonstrate that spectral imaging microscopy can directly detect PCNA ubiquitination by FRET between CFP-Ub and mRFP-PCNA at the level of whole cells.

### Single-Pass Ratiometric Spectral Imaging FRET of PCNA Ubiquitination in Live Cells

The wide separation of CFP and mRFP coupled with spectral imaging therefore potentially allows the direct readout of FRET in living cells. However, there remains a contribution of donor bleed through [CFP^Ex407/λ607^] and direct acceptor excitation [mRFP^Ex407/λ607^] in the raw mRFP FRET signal although this is significantly less than with conventional FRET pairs, such as CFP and YFP.

**Figure 5:**
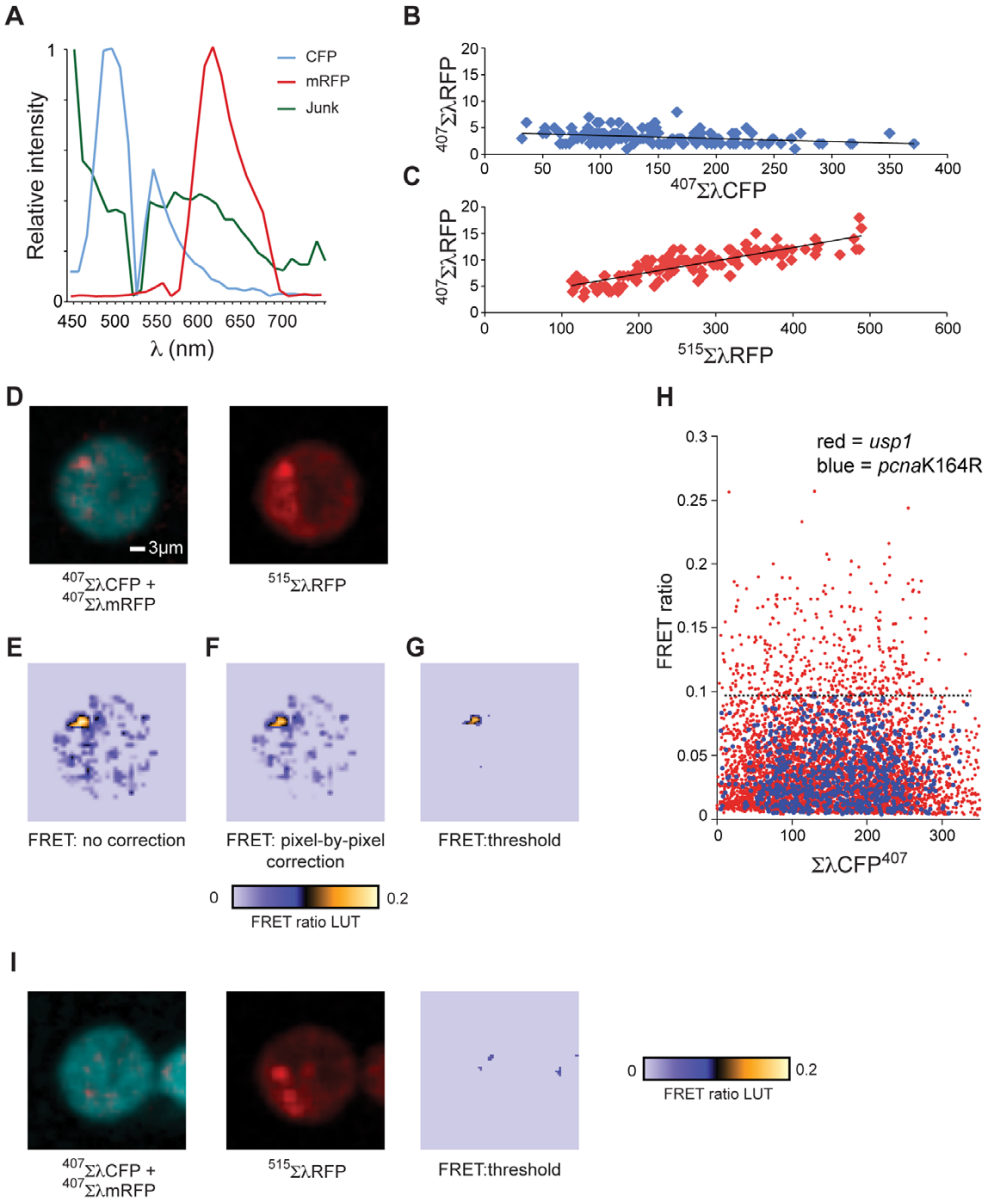
Detection of PCNA ubiquitination *in vivo* by spectral imaging FRET. A. Reference spectra acquired with the Nikon C1-si for CFP (Ex407/ΣλCFP, blue line), mRFP (Ex515/ΣλmRFP, red line) and autofluorescence (‘Junk’) generated by excitation with the 407 nm laser (green line). The dip in the CFP and ‘Junk’ spectra at 515 nm are due to the metal finger protecting the PMT array from the 515 nm laser line. B. Correction of CFP spectral bleedthrough by spectral unmixing. The X axis plots the intensities of the CFP spectral signal (ΣλCFP) following excitation with 407 nm light from whole cells against the Y axis which plots the corresponding mRFP bleedthrough signal, ^407^ΣλmRFP. C. Direct excitation of mRFP by 407 nm light. The X axis plots the intensities of the CFP spectral signal (ΣλCFP) following excitation with 407 nm light from whole cells against the Y axis which plots the corresponding mRFP signal, ΣλmRFP. D. Example of unmixed spectral images of DT40 taken with 407 nm light (left hand panel) and 515 nm light (right hand panel) 20 minutes after UV irradiation through a 3 µm microporous filter. In the left hand image, the unmixed CFP (^407^ΣλCFP) and mRFP (^407^ΣλmRFP) channels are superimposed and ^407^ΣλmRFP enhanced to allow it to be seen in the merge i.e. this image depicts the position of the two signals but not their absolute intensities. E. Uncorrected FRET ratio image (^407^ΣλmRFP∶^407^ΣλCFP). LUT  =  look-up table. F. FRET ratio image determined after pixel-by-pixel correction for direct excitation of mRFP by the 407 nm laser. G. FRET ratio with the threshold applied H. Derivation of the FRET threshold. Scatter plot of ΣλCFP when excited with 407 nm light against the raw FRET ratio derived from the algorithm in D applied to a *usp1* cell field (red) and a *pcna*K164R cell field (blue). The dotted line (at a ratio of 0.1) shows the upper cut off for 99% of the pixels for the *pcna*K164R cell. I. An example of a cell with a focal accumulation of PCNA that does not result in a FRET signal demonstrating the independence of the FRET signal from mRFP bleedthrough. See also legend to Figure 5D.

To remove CFP bleedthrough we employed linear spectral unmixing. Linear spectral unmixing decomposes the spectral information in an image into defined constituent spectra, in this case reference spectra for CFP and mRFP excited at 407 nm and 515 nm. Reference spectra were all collected from control samples imaged under the same conditions as the experimental samples (Figure 5A). We designated the main output channels of the unmixing algorithm ΣλCFP and ΣλmRFP (Figure 5D, Panels [1]–[2]) corresponding to the extracted CFP and mRFP signals respectively. In order to remove any contribution of autofluorescence to the ΣλCFP and ΣλmRFP channels we also added a ‘Junk’ channel to the unmixing algorithm (^407^ΣλmJunk, Figure 5A), which indicates signals produced by background and autofluorescence on excitation by the 407 nm laser. The implementation of the linear unmixing algorithm in the Nikon EZ-C1 software also generates a ‘remainder’ channel, which comprises the fraction of the total signal that was not assigned to one of the defined channels. This provides an indication of the accuracy of the unmixing process and, following unmixing into ΣλCFP, ΣλmRFP and ΣλJunk, accounted for less than 1% of the total signal.

Application of the unmixing algorithm to the spectral image effectively eliminated bleedthrough of CFP fluorescence into the mRFP channel (Figure 5B), as previously described [19]. Following unmixing, we also removed any signal in the ^407^ΣλmRFP channel that also appeared in the ‘Junk’ channel. Figure 5D shows example unmixed images of a wild type DT40 cell with a localised region of FRET signal in its nucleus following UV irradiation through a microporous filter. The raw FRET ratio was calculated by dividing ^407^ΣλmRFP by ^407^ΣλCFP and the resulting ratiometric image is shown in Figure 5E.

This raw FRET image still includes signal arising from direct excitation of mRFP by the 407 nm laser (Figure 5C). We compared two methods that compensated for this signal with leaving the FRET ratio image uncorrected: 1. Pixel-by-pixel correction for the intensity of mRFP when directly excited; 2. the application of a threshold to the ratiometric image based on the genetic controls afforded by the *pcna*K164R and *usp1* cells. In Figure 5F, the direct excitation of RFP is corrected for on a pixel by pixel basis using a paired image of the cell acquired with excitation at 515 nm, the expected bleedthrough being computed using the relationship ^407^ΣλmRFP = 0.025 (^515^ΣλmRFP) +2.3, derived from the experiment shown in Figure 5C. Comparison of Figures 5E and F demonstrates that the contribution of the direct excitation of mRFP by the 407 nm laser is small. This is also demonstrated in Figure 5I, which shows a cell harbouring a focal accumulation of PCNA without any resulting FRET signal. Thus, we suggest that for the purposes of live cell imaging the direct excitation of RFP by the 407 nm laser can be ignored or, if more stringency is required, the threshold applied. Both approaches allow single pass imaging, thereby reducing phototoxicity and the risk of acceptor photobleaching.

### The Kinetics of PCNA Ubiquitination Following Subnuclear UV Irradiation in DT40 Cells

**Figure 6:**
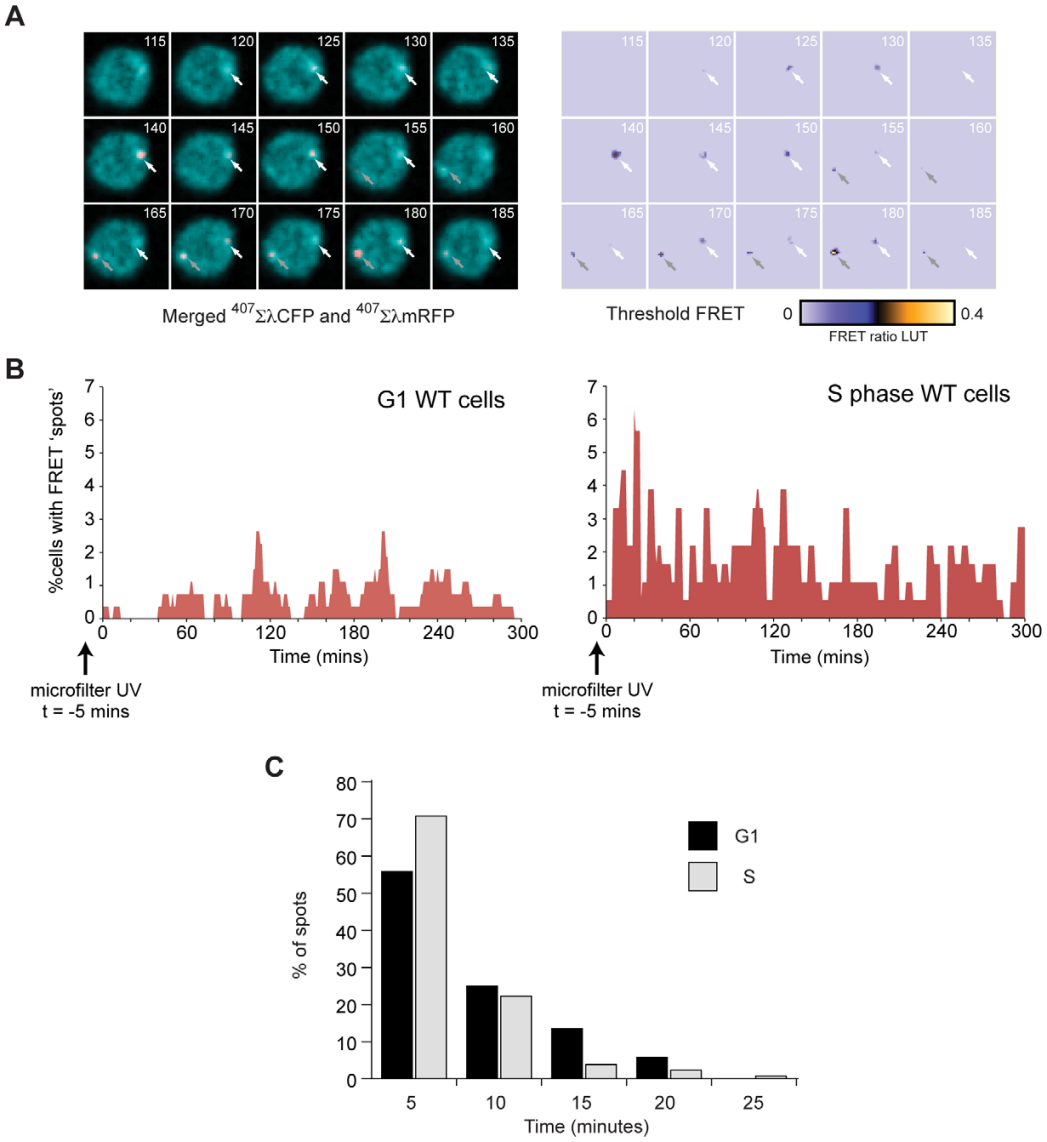
Dynamics of PCNA ubiquitination *in vivo*. A. Consecutive frames from a time-lapse image of a single cell UV irradiated through a 3 µm filter. The number of minutes following microfilter UV irradiation is shown in the top right of each frame. The left hand panel shows the merged CFP (^407^ΣλmRFP) and mRFP (^407^ΣλmRFP) signals (with the ^407^ΣλmRFP enhanced to allow it to be seen against the ^407^ΣλCFP image). The white and grey arrows indicate two independent FRET events. LUT  =  look up table. B. Percentage of cells with FRET spots in DT40 cells irradiated in G1 or in S phase. C. Distribution of FRET spot persistence for cells irradiated in G1 (black bars) or S phase (grey bars).

In order to examine the localised ubiquitination of PCNA we UV irradiated cells through a 3 µm filter [30] prior to live cell imaging at 5 minute intervals using excitation with 407 nm light. Spots of FRET signal were seen throughout the 5 hour imaging window, an example sequence being shown in Figure 6A.

While PCNA ubiquitination has been principally described as an S phase phenomenon, mounting evidence suggests that it can also occur outside of S phase in both G2 and G1, at least in yeasts [31], [32]. To establish whether the phase of cell cycle in which the cells were irradiated influenced the kinetics of PCNA ubiquitination, we synchronised wild type DT40 cells by centrifugal elutriation to produce populations in which >90% cells were in either G1 or S phase. The elutriated populations were UV irradiated through the 3 µm filter and the kinetics of PCNA-ubiquitin FRET signals monitored. Cells irradiated in S phase showed FRET signals at the earliest time point, 5 minutes post irradiation (Figure 6B). In contrast, the proportion of cells irradiated during G1 exhibiting FRET spots was reduced (Figure 6B). Further, significant levels of FRET signal was delayed by about 60 minutes. This is approximately the average length of G1 in DT40, which is 1.5–2 hours at 37°C. Thus, significant PCNA ubiquitination is not seen immediately in G1 and the appearance of the signal at later time points may reflect UV damage being passed through the p53-dependent G1 checkpoint, which is defective in DT40, to S phase.

## Discussion

Here we have described a straightforward method for monitoring the post-translational modification of a protein by monoubiquitination *in vivo* and validated it using genetic controls.

Previously, a method for detecting ubiquitination dynamically in living cells has been described using bioluminescence resonance energy transfer (BRET) [27]. However, BRET does not lend itself to cellular imaging and subcellular localisation. Detection of ubiquitination *in vivo* has also been achieved by the use of fluorescence lifetime imaging FRET and a non-fluorescent acceptor (REACh) [33]. However, this requires specialist lasers and detection equipment. A key feature of the FRET method presented here is its simplicity, utilisation of readily available equipment and a requirement for only a single imaging pass. This facilitates its use in live cell imaging while at the same time minimising phototoxicity and acceptor bleaching.

The success of this method derives from a combination of the use of a widely separated FRET pair with minimal bleedthrough coupled with spectral imaging microscopy. Although CFP and mRFP have not been widely employed in FRET studies, we show here that their performance in terms of FRET transfer efficiency is equal to, or exceeds that of, more conventional FRET pairs such as CFP and YFP.

The very wide spectral separation of the donor and acceptor fluorophores is a potential limitation of the method however as a large portion of the detectable spectrum is used, limiting the ability to combine this technique with other dyes or fluorescent proteins for colocalisation studies with ubiquitinated PCNA. However, this drawback is likely to be overcome by improvements in far-red and infra-red fluorophores [34].

We anticipate that further refinements to this method should make it applicable to the study monoubiquitination of other substrates as well as other protein post-translational modification such as SUMOylation across a range of eukaryotic cells.

## Materials and Methods

### Cell Culture & Synchronisation

DT40 cells were propagated and transfected as previously described [35]. Cell sorting was performed with a MoFlo automated cell sorter (Cytomation). Synchronisation by centrifugal elutriation was performed on a JE-5.0 Elutriation System (Beckman) with a 4 ml chamber as described [36] with elutriation being effected at constant flow with reducing rotor speed.

### DNA Constructs

Expression vectors for CFP-ubiquitin and mRFP-PCNA were constructed using a previously described method in which a HindIII–SalI–NotI three-way ligation into pXPSN results in a SpeI fragment containing the expression cassette [37]. This SpeI fragment was transferred into the SpeI site of either pLoxBsr (in the case of CFP-ubiquitin) or pLoxPuro (in the case of mRFP-PCNA) to provide a selectable marker [38]. To prepare the mRFP-TEV-CFP fusion construct, mRFP with a deleted stop codon was cloned into the SalI and HindIII sites of pGEX6P1 to create pGEX6-mRFP. CFP was amplified with the primers CFPTEVF [5′-AAAAGCTTGAAACCCTATACTTCCAAGGAATGGTGAGCAAGGGCGAGGAGC] and CFPTEVR [5′-AAAAGCGGCCGCCTACTTGTACAGCTCGTCCATGCC] and the resulting products cloned into the HindIII–NotI sites of pGEX6-mRFP. The resulting plasmid pGEX6-mRFP-TEV-CFP drives the bacterial expression of mRFP fused to CFP with a short linker [E-N-L-Y-F-Q-G] incorporating the TEV protease cleavage site.

### Protein Expression and Purification

For expression of the mRFP-TEV-CFP fusion protein, *E. coli* BL21DE3 (Invitrogen) were transformed by heat shock. Following selection with ampicillin, a single colony was grown for 8 hours at 37°C in 2 ml LB, after which 250 µl of this culture was used to inoculate 250 ml LB for overnight growth. 12.5 ml of this culture was inoculated into 500 ml 2XTY and grown to an O.D. of 0.7 at which point protein expression was induced with IPTG at a final concentration of 1 mM. The culture was then grown at 16°C overnight. The bacteria were pelleted and the pellet resuspended and incubated in 40 ml ice cold PBS with 0.1 ml/ml lysozyme, 10 µg/ml DNAse 1, 2 mM DTT and 1 tablet of ‘Complete’ EDTA-free protease inhibitor (Roche) for 10 minutes. Following disruption by sonication the lysate was centrifuged in at Ti45 rotor at 40 krpm for 45 minutes at 4°C. The supernatant was filtered through a 0.45 µm membrane and incubated with rotation with 1 ml glutathione sepharose beads per litre of initial culture, prewashed in PBS, at 4°C overnight. The purified protein was cleaved from the GST tag with 120 µl PreScission protease (GE Healthcare) for 4 hours at 4°C. The protein was dialysed in PBS (with protease inhibitor and 1 mM DTT), concentrated in a Vivaspin column (Vivascience) and flash frozen in aliquots. mRFP and CFP protein was detected in Western blots with anti-mRFP-HRP (Abcam ab34767) at 1∶500 dilution and anti-GFP-HRP (Abcam ab6663) at 1∶1000 dilution, with detection by ECL+ (GE Healthcare).

### Spectrophotometry

Experiments were performed on an LS55 spectrophotometer (Perkin-Elmer) at 22°C with the following settings: excitation slit = 5, emission slit = 15, PMT voltage = 900 V, emission detection range = 450–650 nm, emission step = 1 nm, integration time = 2 s, stirrer speed = 2. The optimal excitation of CFP was determined to be 433 nm and was used for subsequent determinations of FRET. Experiments were performed in TEV cleavage buffer (1 M Tris-HCl pH 7.4, 10 mM EDTA, 10 mM DTT) using 2.7 µg/ml mRFP-TEV-CFP protein in a volume of 20 µl. 10 units of TEV protease (Invitrogen) was added and spectra taken every 30 minutes until the CFP emission peak at 478 nm plateaued.

### Cell Fixation

Approximately 1×10^5^ DT40 cells in PBS were applied to poly-l-lysine (Sigma) treated 22 mm diameter coverslips for 5 minutes in a humidified box following which they were fixed in 4% paraformaldehyde (Alfa Aesar) in PBS for 5 minutes. Cells were mounted in Fluoromount G (Southern Biotech) and sealed under a coverslip.

### Microfilter UV Irradiation

2.5×10^5^ cells were added to a LAB-TEK II Chamber (Nunc) and incubated for 30 minutes at 37°C in 10% CO_2_. For irradiation the medium was removed and replaced with 200 µl of PBS and a 3 µm pore size filter (Millipore) applied to the cell layer. The cells were irradiated with 100 J/m^2^ 265 nm light delivered by a calibrated UV lamp (UVP Inc.) housed in a specially designed shuttered enclosure. The PBS was removed and replaced with pre-warmed RPMI with the usual supplements but without phenol red. The cells were transferred directly to the heated CO_2_ incubation chamber on the microscope.

### Microscopy & Image Analysis

Microscopy was performed with a Nikon C1-si microscope equipped with a Tokai-HIT heated CO_2_ incubation chamber. The objective used was the Plan-Apo Uv-corrected 60x N.A. 1.4. Images were taken in spectral mode with a 10 nm bin size. For illumination of fixed cells the 50 mW 407 nm blue diode laser was used at 10% and 515 nm line of the 40 mW Ar-ion laser (Melles-Griot) also at 10%. For live cell imaging the 407 nm laser was used at 1%. In both cases pixel dwell was set to 9.6 µs. Images were captured every 5 minutes. For photobleaching mRFP, the selected field was scanned with the 515 nm and 561 nm laser lines at full power for 2 minutes. Raw image files were low pass filtered and spectrally unmixed using the high accuracy unmix algorithm in the Nikon EZ-C1 software v3.7 with the reference spectra shown in Figure 5A. Subsequent processing was performed in ImageJ v1.42 [39].
